# Resveratrol-Inspired Benzo[*b*]selenophenes Act as Anti-Oxidants in Yeast

**DOI:** 10.3390/molecules23020507

**Published:** 2018-02-24

**Authors:** Dominika Mániková, Zuzana Šestáková, Jana Rendeková, Danuša Vlasáková, Patrícia Lukáčová, Edgars Paegle, Pavel Arsenyan, Miroslav Chovanec

**Affiliations:** 1 Department of Genetics, Cancer Research Institute, Biomedical Research Center, Slovak Academy of Sciences, Dúbravská Cesta 9, 845 05 Bratislava, Slovakia; zuzana.sestakova@savba.sk (Z.Š.); janka.rendekova@gmail.com (J.R.); exondako@savba.sk (D.V.); lukacovapatricia@gmail.com (P.L.); 2 Department of Medicinal Chemistry, Latvian Institute of Organic Synthesis, Aizkraukles 21, LV-1006 Riga, Latvia; paegle@osi.lv

**Keywords:** benzo[*b*]selenophenes, resveratrol, (supra-)anti-oxidants, yeast cells, reactive oxygen species, redox-modulating molecules, toxicity, DNA strand breakage

## Abstract

Resveratrol is a natural (poly)phenol primarily found in plants protecting them against pathogens, as well as harmful effects of physical and chemical agents. In higher eukaryotic cells and organisms, this compound displays a remarkable range of biological activities, such as anti-oxidant, anti-inflammatory, anti-cancer, anti-aging, cardio- and neuro-protective properties. Here, biological activities of synthetic selenium-containing derivatives of resveratrol—benzo[*b*]selenophenes—have been studied in lower eukaryotes *Saccharomyces cerevisiae*. Their toxicity, as well as DNA damaging and reactive oxygen species (ROS) inducing potencies, manifested through their ability to act as redox active anti-microbial agents, have been examined. We show that some benzo[*b*]selenophenes can kill yeast cells and that the killing effects are not mediated by DNA damage types that can be detected as DNA double-strand breaks. These benzo[*b*]selenophenes could potentially be used as anti-fungal agents, although their concentrations relevant to application in humans need to be further evaluated. In addition, most of the studied benzo[*b*]selenophenes display redox-modulating/anti-oxidant activity (comparable or even higher than that of resveratrol or Trolox) causing a decrease in the intracellular ROS levels in yeast cells. Therefore, after careful re-evaluation in other biological systems these observations might be transferred to humans, where resveratrol-inspired benzo[*b*]selenophenes could be used as supra-anti-oxidant supplements.

## 1. Introduction

Free radicals and other reactive oxygen species (ROS) are historically considered as harmful molecules playing a role, besides aging, in more than 100 human pathologies including cancer, stroke, Alzheimer’s and Parkinson’s diseases, cardiovascular and neurodegenerative diseases, and arthritis and diabetes [1,2]. These molecules contain one or more unpaired electron and hence are highly reactive to compensate this deficiency. A free radical reacts with a non-radical molecule, giving rise to a new radical, thereby starting a chain reaction. Overproduction of free radicals results in oxidative stress (OS) that can damage cellular molecules, such as nucleic acids, lipids, proteins, polyunsaturated fatty acids, and carbohydrates [3].

One of the endogenous ROS—superoxide (O_2_^•−^)—is produced in mitochondria within the oxidative phosphorylation chain and dismutates spontaneously or with the help of superoxide dismutase to hydrogen peroxide (H_2_O_2_) [4]. H_2_O_2_ itself is not very reactive, but in the presence of trace metals like iron or copper, it is converted to extremely reactive hydroxyl radical (OH^•^) in metal-catalysed Haber-Weiss or Fenton reactions [5]. This free radical species is the most reactive one with a half–life of about 10^−9^ s, which leads to a high probability of unavoidable damage on nearby molecules in cells, and hence creating substrates for repair processes [3]. Besides their detrimental effects, low to moderate doses of ROS are beneficial and essential to cellular signalling and response to infectious agents [6]. Therefore, it is important to keep a balance of redox homeostasis with the help of redox-modulators/anti-oxidants within the cell, either by enzymes that convert ROS to less dangerous species (e.g., superoxide dismutase) or by anti-oxidant molecules that scavenge ROS in a non-specific manner (glutathione, thioredoxin). However, the appropriate pro-oxidant/anti-oxidant balance in a normal cell can be shifted towards the pro-oxidant state called OS, when production of oxygen species is increased greatly or when the levels of anti-oxidants are diminished [3]. Exogenous anti-oxidants can be categorized as natural and synthetic. The most commonly used synthetic anti-oxidants in terms of food and pharmacological applications are phenolic compounds, such as butylated hydroxyanisole, butylated hydroxytoluene, tert-butylhydroquinone, and propyl gallate. These compounds have been deeply analyzed for both the toxic and carcinogenic effects, but some new data, coming after a long period of their use, impose some caution. Moreover, there is a growing tendency to have a detailed look at food ingredients among consumers, favouring natural and bio-products, and therefore research should focus on natural and safer sources of anti-oxidants [3]. The most noticeable natural dietary anti-oxidants are vitamin C, tocopherols, carotenoids, and flavonoids.

Mediterranean type of diet has been associated with a significant improvement in health status, reduction in overall mortality, mortality from cardiovascular diseases, incidence of or mortality from cancer, and incidence of Parkinson’s and Alzheimer’s diseases [7]. This diet is rich in vegetables, fruits, legumes, cereals and fish, and includes a moderate intake of red wine during meals. Health benefits attributed to moderate consumption of red wine, contrasting with a relatively high intake of fat, are known as the “French paradox” [8]. Red wine is in fact rich in anti-oxidants—flavonoic and non-flavonoic polyphenols. The most famous wine component is the non-flavonoic one—resveratrol. This phytoalexin is synthetized by plants as a response to a pathogen.

In grapevine, it is produced in the skin of the berries and serves as a protection against the grey mould *Botrytis cinerea* [9]. Besides grapes, *trans*-resveratol can be found in several other plant species including peanut (*Arachis* sp.), trefoil (*Trifolium* sp.), and *Veratrum* sp. [10]. As a potential executor of the benefits of moderate wine consumption, it has attracted a lot of attention in the last 20 years, which resulted in describing its biological effects as anti-oxidant, anti-cancer, anti-diabetic, cardio- and neuro-protective, hormone, gene and enzyme modulator, and even anti-aging agent [11,12].

**Figure 1:**
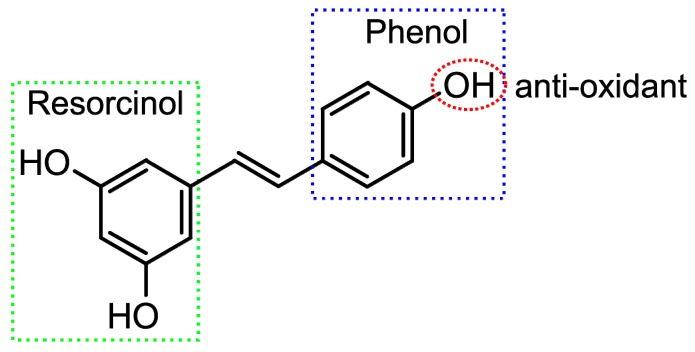
Molecular representation of the anti-oxidant capacity of resveratrol.

Its molecule is composed of a resorcinol and a phenol moiety with several hydroxyl (OH) groups (Figure 1). The anti-oxidant mechanism of this molecule has been widely studied and seems to be dependent on the 4′-OH group, which exhibits a superior H-atom transfer and peroxyl-radical scavenging capacity, in contrast to the resorcinol moiety OH groups [3,13].

Selenium (Se) is an essential trace element required for many physiological processes in the cell and body. It has a hormetic (U-shaped) dose-response associated with cancer—low levels as well as high levels of blood Se are associated with a risk of DNA damage in cells, whereas middle doses are beneficial in terms of cancer prevention [14]. Obviously, less is better in some particular cases, and hence Se supplementation should be administered wisely only to persons who can indeed benefit from it. The effect of Se is not only dose-dependent, but also compound/form-dependent. Se is present in diet in the form of naturally occurring organic compounds (selenomethionine, selenocysteine, Se-methlyselenocysteine), or can be taken as supplement in the form of selenized yeast or inorganic salts (mostly sodium selenite; SeL). The higher toxicity of inorganic SeL compared to the organic molecules [15] could indeed be beneficial to kill selectively cancer cells. As cancer cells are generally more metabolically active and hence have higher intracellular ROS levels, SeL-induced ROS production in these cells can overpass the redox threshold, and thus activate apoptosis in these cells [16]. It has been shown that SeL induces OS in yeast cells, as well as DNA damage in the form of DNA double-strand breaks (DSBs) [15,17,18].

**Figure 2:**
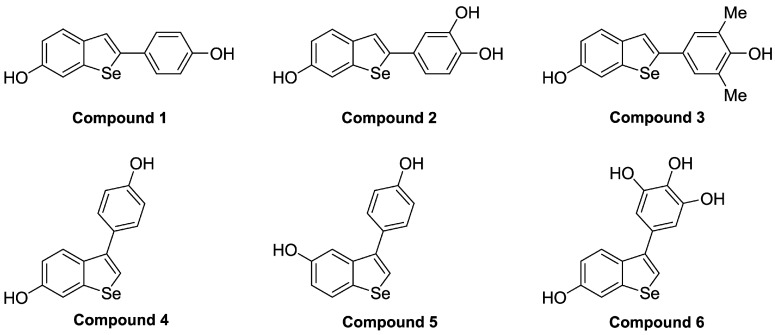
Chemical structures of benzo[*b*]selenophenes used in this study.

The idea of combining the action of resveratrol and Se has already attracted attention of scientists, e.g., Doganay et al. showed that resveratrol suppresses SeL-induced OS and cataract formation in rats [19]. Here, to obtain a powerful anti-oxidant molecule modifying intracellular redox homeostasis, we combined features of resveratrol structure with a Se atom creating resveratrol-inspired polyhydroxybenzo[*b*]selenophenes (Figure 2) [20]. These organoselenium compounds are expected to display a combined biological activity, simultaneously acting as anti-oxidants by removing ROS and pro-oxidants by modifying redox sensitive amino acids [21]. We evaluated the toxicity of these compounds, as well as their ability to induce DNA damage represented by DSBs and to modulate ROS levels in the yeast *Saccharomyces cerevisiae*. We compared their effects with those of resveratrol, SeL and Trolox (6-hydroxy-2,5,7,8-tetramethylchromane-2-carboxylic acid), the last being a water-soluble vitamin E analogue and potent anti-oxidant used as a standard for comparison of radical scavenging capacity in the Trolox equivalent anti-oxidant capacity (TEAC) assay [22,23].

## 2. Results

### 2.1. Toxicity of Benzo[b]selenophenes Measured by Spot Test

**Figure 3:**
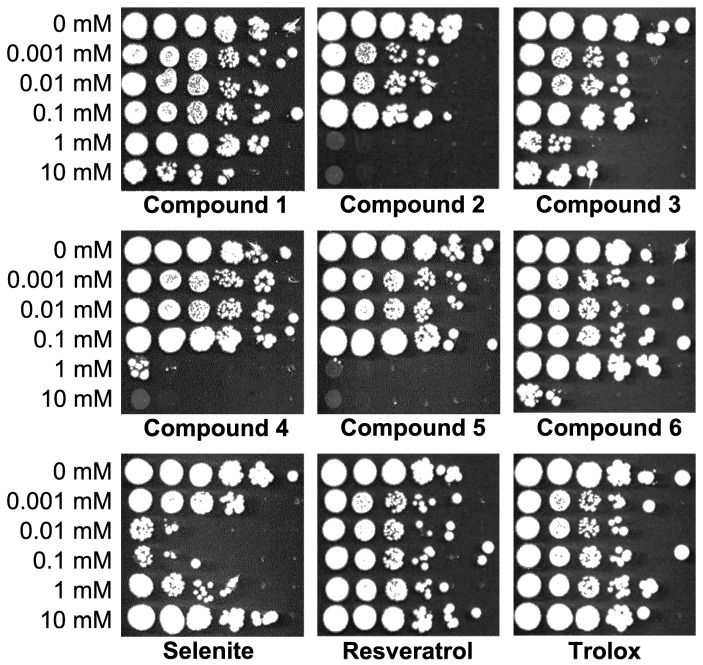
Toxicity of benzo[*b*]selenophenes in *S. cerevisiae* assessed by spot test. Horizontal lines are ten-fold serial dilutions of the yeast cell suspension. Representative experiment is shown.

To get first information on the effects of the studied benzo[*b*]selenophenes in yeast, we tested the viability of yeast cells after treatment with these Se compounds using the semi-quanti-tative spot test. As evident, 2-(3,4-dihydroxyphenyl)6-hydroxybenzo[*b*]selenophene (Compound **2**) and 3-(4-hydroxyphenyl)benzo[*b*]selenophenes (Compound **4** and **5**) exhibited the highest toxicity. They completely inhibited the growth of yeast cells within the concentration range of 1–10 mM (Figure 3). However, the introduction of three OH groups (Compound **6**) led to a decrease of toxicity. This compound achieved a comparable effect only at the highest concentration of 10 mM. On the other hand, Compound **1** and **3** containing only two OH substituents showed only negligible toxicity at the tested dose range. In the case of SeL, the toxicity was dose-independent, with the highest toxicity seen at the moderate concentrations of 0.01–0.1 mM, in accordance with our previous results [17].

### 2.2. Induction of DNA Double-Strand Breaks Measured by Pulsed-Field Gel Electrophoresis

**Figure 4:**
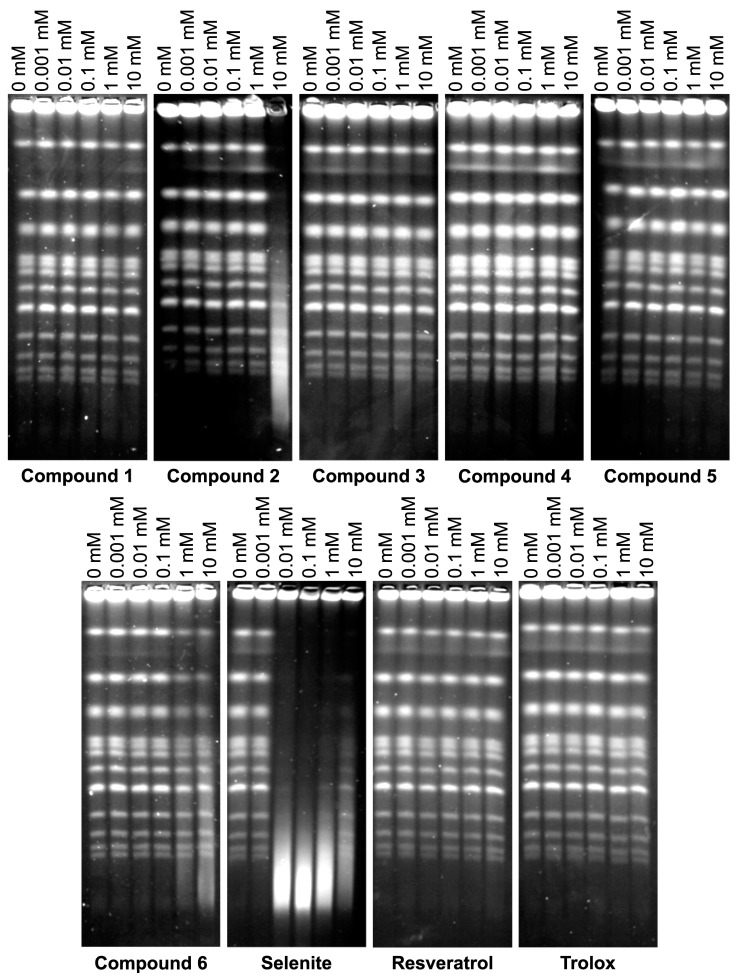
DSBs induction by resveratrol–inspired benzo[*b*]selenophenes in *S. cerevisiae*. Representative gels are shown.

Pulsed-field gel electrophoresis (PFGE) permits visualisation of individual chromosomes in yeast. Apart from other applications, it is commonly used to detect chromosomal DNA damage in terms of DSBs. This DNA damage type is very toxic for cells, as even one unrepaired DSB can cause cell death. DSBs can arise in DNA directly, but also as a consequence of cellular metabolism, e.g., ROS generated as a by-product of mitochondrial respiration can yield DSBs. In this study, we investigated whether resveratrol-inspired benzo[*b*]selenophenes can cause DSBs in yeast cells (Figure 4). As shown previously [17,24], SeL induced DSBs at concentrations equal or higher than 0.01 mM. Of other tested compounds, only Compound **2** and **6** containing three and four OH groups respectively, showed apparent DSB induction, but only at the highest concentration of 10 mM. The remaining compounds did not induce DSBs throughout tested concentration range (0.001–10 mM).

### 2.3. ROS Induction by Benzo[b]selenophenes

As the studied benzo[*b*]selenophenes are inspired by the chemical structure of resveratrol, we investigated the intracellular redox state change in yeast cells being treated by these compounds. After the treatment, a compensated two-colour staining (propidium iodide; PI and 2′,7′-dichlorofluorescin diacetate; DCFDA) and flow cytometry were used to monitor acute toxicity and the ROS levels in living yeast cells, respectively. PI penetrates and stains cells if their membrane integrity is impaired (dead or damaged cells) [25], while DCFDA is hydrolysed in metabolically active cells by intracellular esterases, leading to a fluorescent product. We measured the survival at various concentrations and used the obtained values for calculating LD_50_ (Table 1) using probit analysis [26] and a free online calculator [27]. In accordance with spot test data, the most toxic chemicals in yeast were Compound **2** (2-(dihydroxyphenyl)benzo[*b*]selenophene), **4** and **5** (3-substituted analogues) with a LD_50_ of 1.06 mM, 1.06 mM and 2.05 mM, respectively. It should be noted that the change of OH substituent position in benzo[*b*]selenophene ring from 6 (Compound **4**) to 5 (Compound **5**) led to a considerable increase of LD_50_. Moderate toxicity was observed for Compound **1** with LD_50_ of 4.32 mM and the least toxic were Compound **3** with hindered OH substituent with two methyl groups and Compound **6** (tetrahydroxy benzo[*b*]selenophene) with a LD_50_ of 6.73 mM and 6.16, respectively. Even the highest concentration of resveratrol and Trolox (10 mM) was hardly toxic in yeast with an average surviving fraction being in the range of 90–100%, and therefore LD_50_ for these two compounds could not be estimated.

To compare the effects of the studied Se compounds on the intracellular ROS levels in yeast, a concentration leading to cell survival of 70–90% for each compound was first identified and then used in further experiments (Table 1). The dose used for measuring the ROS levels was always below the LD_50_ concentration, maximally reaching 81% of it. As previously reported [17], SeL significantly affects the ROS status in the cells: at the dose of 50 mM, the ROS levels raised ~2.5-times (258.7 ± 15.6%) compared to the untreated control (Table 1). The known anti-oxidants—resveratrol and Trolox—lowered the ROS levels by about half (49.8 ± 11.8% and 47.3 ± 1.5%, respectively). Of the synthetic Se compounds, 5-hydroxybenzo[*b*]selenophene (Compound **5**) increased the ROS levels to 166.4 ± 28.1%, while its 6-hydroxy substituted analogue (Compound **4**) did not significantly affect them (94.8 ± 16.7%). Other compounds (Compound **1**–**3** and **6**) decreased the ROS levels below that of resveratrol and Trolox. The ROS levels for Compound **1** and **3** were 34.7 ± 3.6% and 41.0 ± 9.5%, respectively. An eight–time decrease in the ROS levels was found after treatment with Compound **2** and **6** (12.0 ± 3.6% and 12.3 ± 1.3%, respectively). It could be concluded that the introduction of more than two OH groups in these compounds allows increased anti-oxidant properties. In addition, combining of the well-known redox catalytic activities of Se and resveratrol in benzo[*b*]selenophenes used herein not always leads to the synergistic effect in terms of anti-oxidant properties (Table 1). Rather, a wide range of the resulting redox-modulating activities has been detected.

## 3. Discussion

There are only a few mentions of hydroxy and alkoxy benzo[*b*]selenophenes in the literature, with most of them being focused on their chemical synthesis, as well as anti-tumor activity [28]. The compounds used herein were synthetized as a part of the study by Paegle et al. [20], in which their structural and electrochemical properties, as well as redox reactions, were assessed using NMR spectroscopy and cyclic voltammetry in MeCN, respectively. In addition, free radical scavenging activity and in vitro cytotoxicity against several cancer cell lines were elucidated. However, no correlation between free radical scavenging activity of these compounds and their cytotoxic effects could be found, indicating that the toxic effects were not necessarily prevented by anti-oxidant properties [20]. We have used a lower eukaryotic organism *S. cerevisiae* as another model system to extend the previous study and to get deeper insights into the mode of action of selected resveratrol-inspired benzo[*b*]selenophenes in vivo. As there is a high evolutionary conservation of factors involved in protection against OS between humans and *S. cerevisiae* [29], deciphering anti-oxidant mechanisms in yeast could shed light on analogous mechanisms in mammalian cells.

The toxicity of benzo[*b*]selenophenes in yeast cells was studied by two different means. First, the spot test as an easy and low cost method was used. In this assay, Compounds **2**, **4** and **5** caused a significant growth inhibition of yeast cells at the concentration range of 1–10 mM (Figure 3). These compounds were also the most toxic in the second assay—flow cytometry after PI staining, where a concentration ≥1 mM considerably increased the number of cells with a damaged membrane or dead cells. Both these methods have their unique features. The spot test is only a qualitative-semi-quantitative method, good enough to determine the concentration range for viability. However, this method does not address the question whether a cell unable to reproduce is still alive or already dead. In contrast, PI staining allows analysis of individual yeast cells and in combination with DCFDA can be used for determining not only the viability, but also the cell vitality (physiological capabilities of a cell—morphological, intracellular or metabolic alterations that result in inability of the cell to reproduce) [25]. In accordance with the in vitro data (basal cytotoxicity test on mouse fibroblasts), the most toxic was Compound **2** and one of the least toxic was Compound **6** [20]. The toxicity of other compounds did not precisely match between mammalian cell lines and yeast cells. The reasons for this discrepancy could be in the cellular constitution of a model organism itself (higher versus lower eukaryote) or difference in treatment conditions (24 h versus 3 h). It was also reported that the introduction of another OH group adjacent to the 4′-OH moiety did not lead to significant changes in the specific anti-proliferative activity towards cancer cell lines, but Compound **2** (two OH groups) was less cytotoxic than Compound **1** (one OH group). In yeast cells, we observed the opposite effect, rather reflecting the response of mouse fibroblasts [20].

In the cell, DCFDA is oxidized in the presence of not only peroxides, but also a broad range of oxidizing reactions present under OS [30]. However, careful attention should be paid to conditions that can yield DCF fluorescence. The cytosolic oxidation of DCFDA to DCF could also depend on the combined effect of Fenton–type reactions and the enzymatic activity of cytochrome c, and hence depends on the relocation of transition metals in redox-active form from lysosomes (vacuoles in yeast) and/or of cytochrome c from mitochondria [31].

We examined the ability of resveratrol-inspired benzo[*b*]selenophenes to modulate the ROS levels in yeast cells by two-colour flow cytometry. We measured fluorescence after staining with DCFDA in PI negative (alive) cells. We confirmed some of the findings by Paegle et al. [20]. Like in their in vitro peroxyl-scavenging assay, the 3-aryl derivative Compound **4** decreased the intracellular ROS levels more effectively than 2-aryl isomer Compound **1** at equimolar concentrations (data not shown). In addition, the presence of an additional 3′-OH group in Compound **2** caused an apparent increase of the anti-oxidant activity compared to Compound **1**, the intracellular ROS levels were 12.5% and 34.7% of the control respectively, despite the higher concentration of Compound **1** used in these experiments. One of the most potent scavengers in vitro was Compound **6** (for 2,2-diphenyl-1-picrylhydrazyl (DPPH) and O_2_^•−^) [20], what is in accordance with our in vivo yeast data showing the lowest ROS levels after 5 mM treatment by this compound (only 12.3% of untreated control).

During the optimization of the ROS detection method, we observed that DMSO on its own can induce ROS, although it does not have the toxic effects. There was only negligible if any change in cell survival in the presence of 5–10% DMSO, whereas the ROS levels increased by 20% (data not shown). To avoid interference between our compounds and DMSO, either 5 or 10% DMSO was used in all samples including untreated controls. In addition, the ROS levels in cells are dependent on the growth phase, being about 18-times higher in the exponentially growing metabolically active cells compared to stationary cells (data not shown), which were obtained directly by processing of overnight cultures.

Another type of dependency that we observed in yeast is that the number of OH groups on the phenyl moiety of the benzo[*b*]selenophene molecule determines its efficacy to act as an anti-oxidant: the more OH groups, the better anti-oxidant. In line with this statement, at nearly identical surviving fraction, Compound **1** (one OH group) displayed about 3-times lower ROS scavenging activity compared to Compound **2** (two OH groups). It is also true in the case of the 3-aryl molecules, where Compounds **4** (one OH group) and **6** (three OH groups), also at comparable survival rate, reduced the ROS levels by about 5% and 88%, respectively (Table 1). In accordance with our already published results [17], SeL increased the intracellular ROS levels. In the previous study, the cells received 10 mM concentration of this inorganic Se compound and using DCFDA we reported an about 2-times increase of the ROS levels compared to untreated cells. Herein, we observed an additional increase of the ROS levels (2.5-times more than in untreated control) at a dose of 50 mM.

Expectedly, the anti-oxidant compounds—resveratrol and Trolox—decreased the ROS levels. A concentration of 10 mM caused about 50% reduction of the intracellular ROS levels compared to untreated control without having a significant impact on cell survival (Table 1). Using various cancer cell lines as well as blood cancer experimental models (lymphoma and leukemia), it has been shown that resveratrol possesses anti-proliferative properties. The mechanisms of action include activation of intrinsic apoptotic pathway, release of cytochrome c from mitochondria, modulation of the proteins belonging to Bcl–2 family, generation of ROS, modulation of p53-dependent pathway, and activation of the extrinsic death receptor pathway [32]. In yeast, previous studies have demonstrated that resveratrol inhibits respiratory growth, while has no effect on cell growth during fermentative phase (before depletion of glucose in the medium). While for resveratrol the main target is the electron transport chain [33], treating yeast cells with Trolox effectively protects them from oxidative damage by scavenging radicals (intracellular O_2_^•−^ and H_2_O_2_) and increasing the activity of the endogenous anti-oxidant enzymes (catalase, superoxide dismutase) [29].

We have shown that the ROS levels decrease significantly after treatment with most of the tested compounds. Further investigation is needed to delineate the underlying mechanism(s). The in vitro scavenging assays performed by Paegle et al. [20] suggest a radical-scavenging activity, but this could be as well a consequence of a disturbance of the cellular respiration in mitochondria, thereby less ROS would be produced. To confirm this assumption, measuring changes in the mitochondrial membrane potential could be performed.

**Figure 5:**
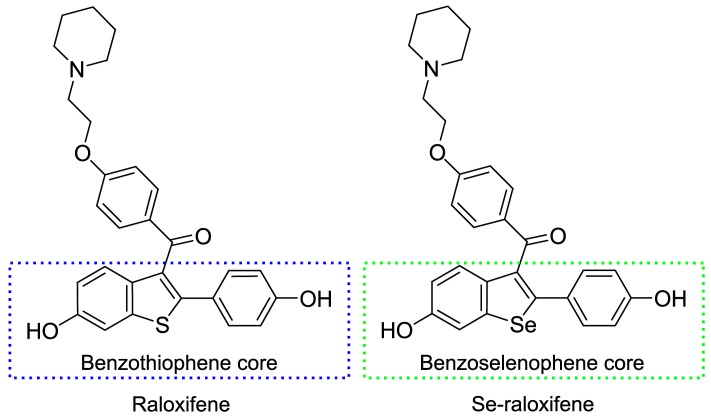
Structures of raloxifene and its Se analogue with the benzothiophene and benzoselenophene cores, respectively. The benzoselenophene core is Compound **1** in our study.

Structural analogy can be found between Compound **1** and the Benzothiophene Core (BTC) of the raloxifene molecule (Figure 5). Raloxifene is a Selective Estrogen Receptor Modulator (SERM) used in clinics for treating osteoporosis in post-menopausal women and decreasing the risk of invasive breast cancer [34,35]. The introduction of a Se atom could intensify this molecule, potentially increasing its redox-modulating activity. Indeed, in vivo experiments showed the ability of the Se-raloxifene to inhibit 4T1 breast cancer cell growth by 30% at a concentration of 15 mg/kg, while raloxifene was ineffective in this context [28]. The partial molecule—BTC—was shown to have chemopreventive potential also in vitro as a transcriptional activator of the Anti-oxidant Responsive Element (ARE)-regulated cytoprotective phase II enzymes, such as the NAD(P)H–dependent quinone oxidoreductase 1 [36]. In our experiments, BTC displayed a LD_50_ value of 8.33 mM (95% CI = 7.22–9.62) and decreased the ROS levels in yeast cells by ~60% (40.7 ± 1.5%, *p*-value 0.0002) at a concentration of 5.67 ± 0.58 mM (data not shown), indicating that this compound in yeast cells is less toxic than the tested benzo[*b*]selenophenes. However, in the study by Paegle et al., this compound had the highest toxicity in mouse fibroblasts [20]. At the moment, we have no satisfactory explanation for this discrepancy besides the difference in model organisms.

Importantly, ROS do not have only a negative impact on cells, but they are also involved in several essential and beneficial processes, such as signalling in immune cells. Considering clinical studies on the relationship between the ROS scavenging agents (anti-oxidants) and diseases prevention, ROS seem to have a hormetic response: too much as well as too little of ROS is detrimental for the cell, and favourable biological effects are achieved only within a hormetic zone. Therefore, supplementation with anti-oxidants should carefully be considered and individual approach based on personal settings to meet the right balance of the ROS levels is rather recommended [37].

## 4. Materials and Methods

### 4.1. Chemicals

Sodium selenite pentahydrate was purchased from Merck, resveratrol and Trolox were obtained from Acros and TCI Europe N. V., respectively. The synthesis of the compounds tested in this study was described by Paegle et al. [20] and their chemical structures are shown in Figure 2. These are as follows: 2-(4-hydroxyphenyl)benzo[*b*]selenophen-6-ol (Compound **1**), 4-(6-hydroxybenzo[*b*]selenophen-2-yl)benzene-1,2-diol (Compound **2**), 2-(4-hydroxy-3,5-dimethylphenyl)benzo[*b*]selenophen-6-ol (Compound **3**), 3-(4-hydroxyphenyl)benzo[*b*]selenophen-6-ol (Compound **4**), 3-(4-hydroxyphenyl)benzo[*b*]selenophen-5-ol (Compound **5**), and 5-(6-hydroxybenzo[*b*]selenophen-3-yl)benzene-1,2,3-triol (Compound **6**).

### 4.2. Cells and Treatment

*Saccharomyces cerevisiae* wild type strain BY4741 (*MATa his3Δ1 len2Δ0 met15Δ0 ura3Δ0*) was obtained from EUROSCARF (http://web.uni-frankfurt.de/fb15/mikro/euroscarf). The cells were grown in YPD medium (1% *w*/*v* yeast extract, 2% *w*/*v* peptone, 2% *w*/*v* glucose, and 2% *w*/*v* agar for plates) overnight at 30 °C with shaking, then used to inoculate fresh medium and cultivated until the next day. The cells were washed with, and resuspended in, phosphate buffer (pH 7.4) (PB) to a concentration of 2 × 10^8^ cells/mL. The treatment by the tested compounds was carried out at room temperature in PB (non-growing conditions) with agitation for 3 h.

### 4.3. Spot Test

The treated cells were serially 10-times diluted in PB (2 × 10^8^–2 × 10^3^ cells/mL) and aliquots (3 μL) were inoculated on YPD agar plates. Cell growth was inspected after 4–5 days’ incubation at 30 °C.

### 4.4. Pulsed-Field Gel Electrophoresis (PFGE)

PFGE experiments were carried out as previously described [24]. After the treatment, 4.5 × 10^7^ cells were washed twice in 50 mM EDTA (pH 8.0) and the resulting pellets were resuspended in a buffer consisting of 10 mM Tris, 20 mM NaCl, 50 mM EDTA (pH 7.5). The cell suspension was equilibrated to 50 °C and 10 μL of lyticase (10 mg/mL in 0.9 M sorbitol, 0.1 M EDTA, 0.1 M Tris, pH 8.0, Sigma, Kawasaki, Japan) was added. The cell suspension was immediately mixed with an equal volume of 2% agarose for PFGE sample preparation (Sigma) and transferred into plug moulds which were kept on ice until plugs completely solidified. The cell wall was degraded by incubating the plugs 1 h at 37 °C in 10 mM Tris, 50 mM EDTA (pH 7.5), supplemented with 0.085 mg/mL lyticase. Next, the plugs were washed with a buffer of 20 mM Tris, 50 mM EDTA (pH 8.0), and subsequently incubated overnight in 100 mM EDTA (pH 8.0), 0.2% *w*/*v* sodium deoxycholate, 1% *w*/*v* sodium lauryl sarcosine, and 1 mg/mL Proteinase K, at 50 °C. Next day, the plugs were washed with a buffer consisting of 20 mM Tris, 50 mM EDTA (pH 8.0) with gentle rolling of the tubes and stored in the same buffer at 4 °C until used. Electrophoresis was performed in TAE buffer (20 mM Tris-acetate and 1 mM EDTA, pH 8.0) using a CHEF-DR^®^ III Variable Angle System (Bio-Rad, Hercules, CA, USA) with constant voltage 4.5 V/cm for 23 h at 14 °C and a switch time of 60–120 s. After electrophoresis, the DNA in gels was stained with 0.5 μg/mL ethidium bromide for 30 min at room temperature and subsequently destained in water containing RNase (4 μg/mL) for 1.5 h. After washing the gel with water, a picture was taken using the MiniBIS Pro Gel Documentation System (DNR Bio-Imaging Systems, Neve Yamin, Israel).

### 4.5. ROS Levels Measurement

#### 4.5.1. Cell Treatment

Yeast cells were inoculated into YPD media and grown overnight. Fresh YPD was inoculated with the overnight culture and the cells were grown until next day. Next day, the cells were counted and concentrated to 2 × 10^8^ cells/mL in PB. Treatment with the compounds was carried out at room temperature for 3 h with constant shaking. As all stocks of the organic compounds were prepared by dissolving them in DMSO, we added DMSO also to sodium selenite as well as control to have appropriate DMSO content in all samples. After the treatment, the cells were washed with, and resuspended in PB. The cells were diluted 10 times and used for flow cytometry analysis.

#### 4.5.2. Staining and Analysis

The staining was performed as previously published [17]. The cells were added to 10 μM 2′,7′-dichlorofluorescin diacetate (DCFDA-Sigma). Staining was performed at 30 °C in the dark for 1 h. Afterwards, the dye was washed away and the cell pellets were resuspended in ice-cold PB. To discriminate between dead and living cells, PI (7.5 μg from stock 1 mg/mL) was added to each sample (500 μL) before scoring 20,000 cells on a BD FACS Canto II flow cytometer (BD Biosciences, Franklin Lakes, NJ, USA). FITC (530/30 nm) and PE (585/40) filters were used for DCFDA and PI fluorescence, respectively. As we used fluorescence compensation, we were able to exclude dead cells (PI^+^) and hence measure the ROS levels in only living cells (PI^−^). To compare the effect of the compounds with each other, we measured the ROS levels at their concentrations that gave the survival of about 70–90% of the control. FCS Express 4 (De Novo Software, Glendale, CA, USA) was used to score DCFDA fluorescence ratio of medians in samples against non-treated control. Statistical significance of at least three individual experiments was assessed using one sample Student’s *t*-test [38].

## 5. Conclusions

Biological activity of the selected group of benzo[*b*]selenophenes, synthesis of which was inspired by the structure of resveratrol, was examined herein in terms of the toxic effects and the ability to induce DNA damage and ROS. Although no straightforward correlation could be found among the studied end points, nevertheless our in vivo data brought some conclusions. First, some benzo[*b*]selenophenes exhibit the toxic effects against yeast cells, and therefore they potentially could be used as anti-fungal agents. Second, a majority of benzo[*b*]selenophenes demonstrate anti-oxidant activity suggesting their use as anti-oxidant supplements in human diet, particularly those with no toxic and genotoxic effects at concentrations relevant to human biology (e.g., Compound **6**). We propose that approach based on combining features of well-characterized molecules with redox-modulating catalytic activity, providing humans with highly beneficial effects, could be useful to design supra-supplements for humans to prevent them from various pathologies.

## Figures and Tables

**Table 1:** LD_50_ with 95% confidence interval (CI) and the ROS levels at 70–90% survival.

Compound	LD_50_ (95% CI) (mM)	ROS Measurement			
		Concentration (mM) ^1,2^	Survival (%) ^1^	ROS Levels (% of Control) ^1^	*p*-Value
1	4.32 (3.32–5.61)	1.93 ± 0.19	82.8 ± 4.0	34.7 ± 3.6	<0.0001
2	1.06 (0.94–1.20)	0.68 ± 0.05	85.1 ± 3.4	12.0 ± 3.6	<0.0001
3	6.73 (6.00–7.54)	5	87.0 ± 3.8	41.0 ± 9.5	0.0086
4	2.05 (1.27–3.31)	0.5	78.5 ± 4.1	94.8 ± 16.7	0.5700
5	1.06 (0.78–1.44)	0.31 ± 0.02	83.9 ± 4.2	166.4 ± 28.1	0.0062
6	6.16 (5.64–6.74)	5	84.3 ± 1.9	12.3 ± 1.3	0.0002
Selenite	73.48 (59.60–90.59)	50	80.7 ± 3.5	258.7 ± 15.6	0.0032
Resveratrol	n/a ^3^	10	94.4 ± 5.9	49.8 ± 11.8	0.0034
Trolox	n/a ^3^	10	104.2 ± 3.3	47.3 ± 1.5	0.0003

^1^ The values are expressed as average ± standard deviation (SD); ^2^ Average concentration of at least three individual experiments in which 70–90% survival was achieved; ^3^ n/a—not applicable; for resveratrol and Trolox, we could not achieve a higher working concentration with available 100 mM stocks to keep the DMSO content at a maximum of 10%. Survival at 10 mM concentration was still too high to be able to calculate LD_50_ for these two compounds.
